# Retraction Note: CDK4 and miR-15a comprise an abnormal automodulatory feedback loop stimulating the pathogenesis and inducing chemotherapy resistance in nasopharyngeal carcinoma

**DOI:** 10.1186/s12885-021-08000-6

**Published:** 2021-03-15

**Authors:** Zhen Liu, Chao Cheng, Xiaojun Luo, Qiong Xia, Yejie Zhang, Xiaobing Long, Qingping Jiang, Weiyi Fang

**Affiliations:** 1 Third Affiliated Hospital of Guangzhou Medical University, Guangzhou, 510150 P.R. China; 2 Cancer Research Institute, Southern Medical University, Guangzhou, 510515 China; 3 Cancer Center, Traditional Chinese Medicine-Integrated Hospital, Southern Medical University, Guangzhou, 510315 Guangdong China; 4 Department of Pathology, Basic School of Guangzhou Medical University, Guangzhou, 510182 China; 5 Pediatric Center of Zhujiang Hospital, Southern Medical University, Guangzhou, 510282 P.R. China; 6 Otorhinolaryngology of Zhujiang Hospital, Southern Medical University, Guangzhou, 510282 P.R. China

**Retraction Note: BMC Cancer (2016) 16:238**

**https://doi.org/10.1186/s12885-016-2277-2**

The Editor has retracted this article because of irregularities in two of the Figures. Specifically:

In Fig. 5e the HONE1 mouse appears to be the same as the shENO1+DDP mouse in Fig. 7b in [1] but the excised tumours shown on the two photos appear to be different

In Fig. 6a the 5-8F/E2F1 and HONE1/E2F1 panels appear to be the same

The Editor therefore no longer has confidence in the results and conclusions presented. Zhen Liu, Chao Cheng, Qingping Jiang and Weiyi Fang do not agree with this retraction. Xiaojun Luo, Qiong Xia, Yejie Zhang and Xiaobing Long have not responded to correspondence from the publisher about this retraction.
